# Exploring the Therapeutic Implications of Co-Targeting the EGFR and Spindle Assembly Checkpoint Pathways in Oral Cancer

**DOI:** 10.3390/pharmaceutics16091196

**Published:** 2024-09-11

**Authors:** Mafalda Calheiros-Lobo, João P. N. Silva, Bárbara Pinto, Luís Monteiro, Patrícia M. A. Silva, Hassan Bousbaa

**Affiliations:** 1 UNIPRO—Oral Pathology and Rehabilitation Research Unit, University Institute of Health Sciences (IUCS), Cooperativa de Ensino Superior Politécnico e Universitário (CESPU), Rua Central de Gandra, 1317, 4585-116 Gandra, Portugal; mafalda.duarte@iucs.cespu.pt (M.C.-L.); joaosilva_06@hotmail.com (J.P.N.S.); barbara.fernandes.15@gmail.com (B.P.); luis.monteiro@iucs.cespu.pt (L.M.); 2 Medicine and Oral Surgery Department, University Institute of Health Sciences—CESPU (IUCS-CESPU), 4585-116 Gandra, Portugal; 3 Associate Laboratory i4HB, Institute for Health and Bioeconomy, University Institute of Health Sciences—CESPU, 4585-116 Gandra, Portugal; 4 UCIBIO—Applied Molecular Biosciences Unit, Translational Toxicology Research Laboratory, University Institute of Health Sciences (1H-TOXRUN, IUCS-CESPU), 4585-116 Gandra, Portugal

**Keywords:** EGFR, MPS1, targeted therapy, preclinical assays, clinical trials, head and neck cancer

## Abstract

Head and neck cancer (HNC), the sixth most common cancer worldwide, is increasing in incidence, with oral squamous cell carcinoma (OSCC) as the predominant subtype. OSCC mainly affects middle-aged to elderly males, often occurring on the posterior lateral border of the tongue, leading to significant disfigurement and functional impairments, such as swallowing and speech difficulties. Despite advancements in understanding OSCC’s genetic and epigenetic variations, survival rates for advanced stages remain low, highlighting the need for new treatment options. Primary treatment includes surgery, often combined with radiotherapy (RT) and chemotherapy (CT). Cetuximab-based chemotherapy, targeting the overexpressed epidermal growth factor receptor (EGFR) in 80–90% of HNCs, is commonly used but correlates with poor prognosis. Additionally, monopolar spindle 1 (MPS1), a spindle assembly checkpoint (SAC) component, is a significant target due to its role in genomic fidelity during mitosis and its overexpression in several cancers. This review explores EGFR and MPS1 as therapeutic targets in HNC, analyzing their molecular mechanisms and the effects of their inhibition on cancer cells. It also highlights the promise of combinatorial approaches, such as microtubule-targeting agents (MTAs) and antimitotic agents, in improving HNC therapies, patient outcomes, and survival rates.

## 1. Introduction

Head and neck cancer (HNC) is the sixth most common cancer subsite globally, with more than 890,000 new cases and 450,000 deaths reported in 2022 [1]. The incidence of HNC is on the rise, and it is estimated to increase around 30% annually by 2030 [2,3]. Oral squamous cell carcinoma (OSCC) is the most widespread histological subtype of HNC, representing approximately 90% of all cases, affecting more males than females, with middle-aged to elderly men being the most susceptible [3]. The posterior lateral border of the tongue exhibits the highest incidence of OSCC, approximately 50% of all cases, followed by occurrences on the floor of the mouth, the soft palate, the gingiva, the buccal mucosa, and the hard palate [4]. This cancer leads to disfigurement and functional impairments, including difficulties with swallowing, speech, and taste, which significantly impact the quality of life for patients [5]. OSCC is a heterogeneous neoplasm with an arrangement of genetic and epigenetic variations linked with different risk factors [6]. These traits provide cancer cells with several advantages in division and survival, such as growth factor-independent spread, resistance to apoptosis, and an increased capability to surpass extracellular matrix barriers and invade adjacent or distant tissues, contributing to aggressiveness and unsuccessful treatment [7]. Clinically, it is characterized by red and white or solely red lesions with a slightly uneven surface and distinct borders. Ulceration is a common symptom of OSCC, presenting with an irregular floor and margins and a hard texture upon palpation [8]. 

The treatment approach for OSCC often involves a combination of modalities such as surgery, radiotherapy (RT), and chemotherapy (CT). The specific treatment plan depends on factors such as the location and stage of the disease, as well as the patient’s overall health and preferences. For early and advanced stages of OSCC, surgery remains the primary treatment, which may be combined with RT and CT for patients with a high risk of recurrence or with pathological adverse features such as positive or close margins, perineural invasion, vascular invasion, and lymphatic invasion [9]. For pharynx and larynx cancers, primary chemoradiation is often the preferred approach, particularly for HPV-positive tumors [10]. OSCC primarily metastasizes to ipsilateral lymph nodes in the neck through lymphatic drainage but can also invade contralateral or bilateral lymph nodes. Typical metastatic sites for OSCC include the lungs, bones, and liver [11].The overall survival for patients with advanced stages of HNSCC is quite low and new treatment options need to be investigated [12]. 

Cetuximab-based chemotherapy is one of the most widely used treatments for HNSCC, a monoclonal antibody that targets the epidermal growth factor receptor (EGFR), a receptor tyrosine kinase (RTK) within the HER/ErbB family, which includes HER2–4 [13,14]. In head and neck cancers, EGFR is overexpressed in 80–90% of cases and is linked to poor prognosis and less favorable treatment outcomes [10]. The signaling network involving EGFR is complex and involves several individual components, as well as overlap with other pathways [15]. The activation of RTK signaling pathways in human cancers is attributed to various events, including ligand or receptor overexpression, aberrant ligand binding, and gene rearrangements. These events ultimately result in enhanced tumor cell migration, survival, and proliferation [16]. Although early results were promising and led to the clinical use of Cetuximab, one of the first approved targeted therapies for solid tumors, both intrinsic and acquired resistance frequently develop, negatively impacting treatment outcomes [17].

To prevent the development of abnormal cells, quality control mechanisms, called checkpoints, are established at various phases of the cell cycle, both at interphase and mitosis [18]. The spindle assembly checkpoint (SAC) keeps the cell arrested in mitosis until all chromosomes form stable bipolar attachments to mitotic spindle microtubules and become aligned at the equatorial midzone [19]. Any defect in this path causes premature exit from mitosis, without proper chromosome attachment and biorientation, leading to mis-segregation and, consequently, to the formation of aneuploid cells [20]. SAC components have garnered significant interest as promising microtubule-independent targets, leading to the development of inhibitors against many of these components, with encouraging results in preclinical studies that have advanced to clinical trials for cancer therapy [21].

Monopolar spindle 1 (MPS1), also referred to as threonine tyrosine kinase (TTK), is an essential component of the SAC pathway [22]. MPS1 is overexpressed in different cancer types, including breast cancer, hepatocellular carcinoma, pancreatic cancer, gastric cancer, and endometrial cancer, and is correlated with poor patient prognosis [23,24,25,26]. In addition, in breast cancer cell lines, the reduction in MPS1 expression led to decreased cell viability, making it a promising cancer therapeutic target [27]. 

As mentioned above, EGFR is overexpressed in a significant majority of HNSCC cases and is associated with aggressive tumor behavior and resistance to conventional therapies. Similarly, MPS1 is overexpressed and its overexpression correlates with poor prognosis in several cancers. Therefore, simultaneously targeting the EGFR pathway and the MPS1-mediated SAC pathway appears to be a logical approach to overcoming HNSCC resistance to conventional treatments.

In this review, we explored the significance of EGFR and MPS1 as therapeutic targets in head and neck cancer. Our analysis covered their roles in both preclinical and clinical stages. We explored their molecular mechanisms, the impact of their inhibition on cancer cells, and the current status of therapies targeting these proteins, both alone and in combination. By accessing data from preclinical studies and clinical trials, we provided insights into the potential benefits and challenges of targeting EGFR and MPS1 in the treatment of HNC. This comprehensive overview underscores the promise of these targets in developing more effective and specific therapies for HNC patients. 

## 2. Targeting EGFR for Head and Neck Cancer Treatment

**Figure 1:**
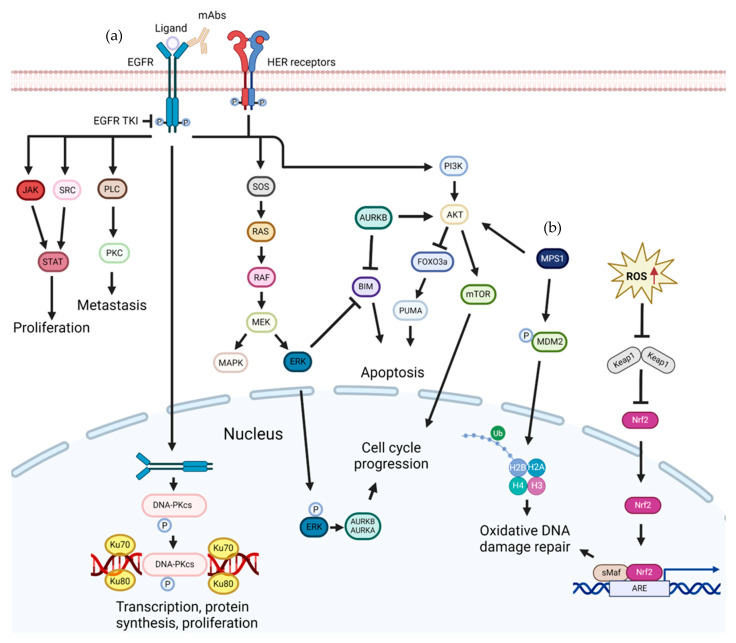
EGFR/HER (**a**) and MPS1 (**b**) signaling pathways. EGFR signaling activates the RAS/RAF/mitogen-activated protein kinase pathway, PI3K/Akt pathway, PLC pathway, signal transducers and activators of transcription pathway, and Src kinase pathway, while MPS1 is involved in the Akt/mTOR pathway and in the activation of oxidative DNA damage repair. Abbreviations: ARE, adenylate-uridylate-rich elements; Aurk A, aurora kinase A; Aurk B, aurora kinase B; BIM, B-cell lymphoma 2 interacting mediator of cell death; DNA, deoxyribonucleic acid; DNA-PKs, DNA-dependent protein kinases; EGFR, epidermal growth factor receptor; ERK, extracellular signal-regulated kinase; FOXO3a, forkhead box O3a; H, histone; HER, human epidermal growth factor receptor; mAbs, monoclonal antibodies; MAPK, mitogen-activated protein kinase; MDM2, mouse double minute 2 homolog; MEK, mitogen-activated protein kinase kinase; MPS1, monopolar spindle 1; mTOR, mammalian target of rapamycin; Nrf2, nuclear factor erythroid 2–related factor 2; P, phosphate; PI3K, phosphatidylinositol-3 kinase; PKC, protein kinase C; PLC, phospholipase Cγ; PUMA, p53 upregulated modulator of apoptosis; RAF, rapidly accelerated fibrosarcoma; RAS, rat sarcoma virus; ROS, reactive oxygen species; sMaf, small musculoaponeurotic fibrosarcoma; SOS, son of sevenless; TKI, tyrosine kinase inhibitor; Ub, ubiquitin.

EGFR is a 170 kDa transmembrane glycoprotein composed of a single polypeptide chain of 1186 amino acids that belongs to the RTK family [28]. The structure of EGFR can be divided into three domains: an extracellular ligand-binding domain, a transmembrane domain, and an intracellular domain. The extracellular domain of EGFR consists of 621 amino acids and is responsible for binding to ligands such as epidermal growth factor. The transmembrane domain of EGFR contains 23 hydrophobic amino acids that anchor the protein in the plasma membrane. The intracellular domain of EGFR contains 542 amino acids and is composed of a tyrosine kinase domain. This domain is responsible for initiating intracellular signaling cascades upon ligand binding. EGFR has several phosphorylation sites at Y845, Y974, Y992, Y1045, Y1068, Y1086, Y1101, Y1148, and Y1173 [29]. Some of these sites serve as docking sites for intracellular signaling proteins [30]. In addition, Y845 and Y1101 are two oncogenic signal-activated transphosphorylation sites that are phosphorylated by other tyrosine kinases, leading to the activation of downstream signaling pathways that promote cell growth and proliferation (Figure 1a) [31,32]. 

A protein binding to specific phosphotyrosine residues in EGFR initiates intracellular signaling through the following pathways: RAS/RAF/mitogen-activated protein kinase pathway, phosphatidylinositol 3-kinase (PI3K)/Akt pathway, phospholipase Cγ pathway, signal transducers and activators of transcription pathway, Src kinase pathway, endocytic pathway, stress pathway, and autophagy pathway (Figure 1a) [33].

The primary function of EGFR is to regulate cellular processes such as growth, proliferation, differentiation, and survival in various cell types, including epithelial, glial, and neuronal cells [34]. In epithelial cells, the EGFR plays a vital role in regulating cell growth, differentiation, and migration [35]. Its expression is particularly prominent in the epidermal basal layer and the outer root sheath of hair cells. Here, it plays an important role in promoting keratinocyte cell migration and differentiation, as well as follicular cell development [36]. In neurons, EGFR signaling pathways are involved in the regulation of migration and neurodegeneration. Mutations in EGFR can cause glioma-like tumors, highlighting its importance in regulating cellular processes that impact cancer development [37]. The expression and function of EGFR are regulated by receptor downregulation or dephosphorylation. However, abnormal expression of EGFR is observed in human epithelial cancers, including HNSCCs [38]. Overexpression of EGFR is associated with poor cancer prognosis, likely due to its role as a signal for cell proliferation [39]. 

Currently, two main strategies are employed to target EGFR with distinct mechanisms [40]. These include inhibiting the tyrosine kinase domain activity (intracellular) with small molecules and blocking extracellular ligand binding by using monoclonal antibodies (mAbs). mAbs that target EGFR selectively recognize the EGFR protein and bind to its extracellular domain competing with the receptor binding of ligands and blocking ligand-induced activation of EGFR tyrosine kinase activity, resulting in the internalization and degradation of the antibody–receptor complex [41]. This leads to tumor cell death through direct inhibition of EGFR signaling. Additionally, these antibodies mediate tumor cell elimination via an indirect mechanism known as antibody-dependent cell-mediated cytotoxicity (ADCC), which involves the activation of natural killer (NK) cells [42,43]. EGFR tyrosine kinase inhibitors (TKIs) compete in a reversible manner with ATP and bind to the intracellular domain of EGFR tyrosine kinase. This binding inhibits the autophosphorylation of EGFR and downstream signaling pathways. In addition to blocking EGFR activity, these inhibitors can also inhibit other growth factor receptor tyrosine kinases, including vascular endothelial growth factor receptor (VEGFR), an important regulator of angiogenesis, which is critical for tumor growth and progression. By inhibiting VEGFR, EGFR TKIs can hinder the formation of new blood vessels and limit nutrient and oxygen supply to tumor cells [44]. 

Until the recent FDA approval of immunotherapies, Cetuximab, a human–murine chimeric anti-EGFR immunoglobulin G-1 antibody approved in 2006, has been the only targeted drug approved for locally advanced HNC (LA-HNC) (HPV-positive and -negative subtypes) in combination with radiotherapy (RT) and as a monotherapy for recurrent or metastatic (R/M) cancer after failure of platinum-based therapies [45]. However, Cetuximab and other therapies designed to target EGFR have limited efficacy mostly due to cancer cells acquiring resistance through alterations in EGFR or downstream EGFR signaling pathways such as PI3K/AKT [46,47,48,49]. For instance, as a monotherapy, only 13% of patients showed responses, which suggests some form of intrinsic resistance, while several patients with positive tumor response acquired resistance to Cetuximab, leading to recurrences [50].

Even though of all EGFR-targeting drugs, only Cetuximab has been approved by the FDA for HNC treatment, the use of Nimotuzumab in HNC has been approved in several countries [51,52]. However, other EGFR-targeting inhibitors, including Gefitinib, Erlotinib, Lapatinib, Afatinib, Panitumumab, Zalutumumab, Poziotinib, Duligotuzumab, Patritumab, and Dacomitinib, have been investigated in clinical trials for HNC therapy (Table 1). Indeed, these studies frequently encompass patients with advanced or recurrent HNSCC who exhibit suboptimal responses to conventional therapeutic modalities or possess restricted therapeutic alternatives. 

For instance, Gefitinib, a reversible EGFR TKI, was investigated in a phase III trial for the treatment of R/M HNSCC. Unfortunately, the study did not show any significant improvement in overall survival (OS) compared to Methotrexate, and as a result, Gefitinib is unlikely to be used clinically for HNSCC [53]. However, it is important to note that Gefitinib is still FDA-approved for the treatment of locally advanced or metastatic NSCLC with EGFR mutations. It has been shown to significantly improve PFS and overall response rate (ORR) in these patients compared to standard chemotherapy [54]. Therefore, Gefitinib remains an important treatment option for NSCLC patients with EGFR mutations in the tyrosine kinase domain (TMD), specifically, those with exon 19 deletions or a substitution in codon 858 of a leucine for an arginine in exon 21 [50,53]. 

A systematic review found that exon 19 deletions and the L858R substitution comprise approximately 25% (22% and 2.5%, respectively) of all EGFR mutations in HNSCC compared to approximately 50% in NSCLC [55,56]. However, mutations in the TMD are observed in 0 to 8% of HNSCC patients, depending on ethnicity and region/country. Moreover, mutations in exon 20 seem to be more frequent [57]. In addition, two studies showed that EGFR mutation status was not predictive of Gefitinib response in patients with HNC. However, the number of patients assessed was limited in both studies (2 out of 15 and 3 out of 19 patients presenting EGFR mutations) [58,59]. Nonetheless, in one of the studies, patients with somatic EGFR mutations showed better time to progression and survival than patients without these mutations, even if not statistically significant [59]. However, another report assessing somatic mutations in EGFR in patients who were responsive to Gefitinib or Erlotinib treatment showed that none of the eight patients analyzed had any EGFR mutation [60].

Furthermore, contrary to NSCLC, in HNSCC, Gefitinib resistance is usually not acquired through EGFR mutations [61]. Instead, HNSCC cells develop resistance by alterations downstream of EGFR overcoming EGFR signaling dependence [62]. Moreover, EGFR TMD mutations in NSCLC are observed in adenocarcinomas and never-smokers at a higher frequency while in HNSCC these mutations can occur in squamous cell carcinomas and smokers [63,64]. Thus, other tumor characteristics might influence the predictive factors for Gefitinib treatment response. However, in recurrent HNSCC, Gefitinib showed equivalent efficacy to Methotrexate both alone and in combination with Fluorouracil and it seemed to improve the quality of life in these patients [65,66,67]. Thus, it is possible that Gefitinib treatment might be more interesting for recurrent HNSCC than for patients with mutated EGFR. Nonetheless, studies focusing on HNSCC patients with exon 19 deletions and L858R substitution treated with Gefitinib should be conducted to clarify once and for all their predictive potential in HNSCC.

Erlotinib is also a reversible EGFR-binding TKI that showed good tolerability and some antitumor activity in phase II studies both as monotherapy and in combination with Cisplatin in patients with R/M HNSCC. However, randomized phase II trials did not show any significant improvement in recurrence rate or PFS in patients with R/M HNSCC, and phase II trials in patients with EGFR variant III mutations also showed no significant difference between Erlotinib-treated and control patients [68,69,70]. As of now, no successful phase III studies of Erlotinib have been completed in the treatment of HNSCC. 

Lapatinib is a reversible dual TKI that targets EGFR and HER2 and inhibits tumor progression. The drug has received FDA approval for the treatment of metastatic breast cancer in combination with Capecitabine [71]. Phase II clinical trials have demonstrated Lapatinib’s potential as a therapeutic agent in previously untreated patients with HNSCC. Results showed an ORR of 17% compared to 0% for placebo [72]. Another phase II trial conducted in patients with locally advanced HNSCC demonstrated that Lapatinib with RT was well tolerated and resulted in increased complete response rates of 53% compared to 36% with placebo six months post-treatment [73]. However, a subsequent phase III study revealed that adjuvant Lapatinib therapy in combination with long-term concurrent chemoradiotherapy did not improve outcomes in patients with surgically treated high-risk HNSCC. Moreover, the treatment was associated with additional toxicity. Therefore, further research is needed to evaluate the safety and efficacy of Lapatinib in patients with HNSCC [74]. 

As a potent and irreversible pan-HER inhibitor, Afatinib binds to and inhibits the activities of EGFR, HER2, and HER4 [75]. A phase II clinical trial conducted in patients with R/M HNSCC who failed prior platinum-based chemotherapy showed that Afatinib was comparable to Cetuximab in terms of tumor size reduction, ORR, and disease control rates [76]. Moreover, Afatinib as maintenance therapy after platinum-based chemotherapy was associated with improvements in PFS and had a manageable safety profile. The PFS achieved with Afatinib was 2.6 months compared to 1.7 months for the Methotrexate group [77]. Further, phase III studies have demonstrated that Afatinib therapy in patients with R/M HNSCC who are 65 years of age or older did not have worse safety profiles compared to Methotrexate therapy alone. These positive results suggest that Afatinib may have the potential to be approved for clinical use in HNSCC [78]. 

Dacomitinib is a small molecule that inhibits EGFR, HER2, and HER4 by irreversibly binding to them [79]. The efficacy of Dacomitinib as a first-line treatment for R/M HNSCC has been investigated in only one phase II clinical trial. The study reported a median PFS of 2.8 months and OS of 9.3 months [80]. However, these findings are insufficient to draw definitive conclusions about the efficacy of Dacomitinib in treating HNSCC. Nonetheless, further randomized and controlled studies are needed to assess the safety and efficacy of EGFR TKIs as a treatment option for HNSCC. 

Even though the targeting of EGFR with Cetuximab in HNC has proven to be quite successful mostly when in combination with taxanes, a class of microtubule-targeting agents (MTAs), 5-Fluorouracil, and Cisplatin or radiotherapy, resistance and tumor recurrence can occur [81,82]. This need to overcome resistance and enhance therapeutic efficacy has led to extensive research on antimitotic drugs. Thus, other combinatorial approaches with EGFR should probably be explored.

**Table 1:** Clinical trials targeting EGFR alone or in combination for HNSCC treatment (from 2002 to 2024).

Drug	Disease	Intervention	Phase	Results	NCT/References
Cetuximab, EGFR Monoclonal Antibody	Dysplastic lesions of the upper aerodigestive tract	Cetuximab	II	Cetuximab-treated patients showed a higher reduction in grade of dysplasia, with 4 of 12 patients showing no dysplasia after treatment and none of the 5 patients in the observation groups achieving complete resolution.	NCT00894413 [83]
	LA HNSCC	Cetuximab		Cetuximab improved the suppressive phenotypes of FcγR-bearing myeloid cells, leading to better clinical outcomes.	NCT01218048 [84]
	R/M SGC	Cetuximab	II	Stable disease for ≥6 months was observed in half of the treated R/M SGC patients. In particular, all patients with adenoid cystic carcinoma achieved stable disease for ≥6 months.	[85]
	LA HNSCC	Cetuximab	II	Twelve weeks of cetuximab maintenance improved outcomes in the first year but this improvement was not observed after 2 years.	[86]
	R/M HNSCC	Cetuximab	II	No therapeutic benefit was observed with increased dose of Cetuximab	[87]
	R/M HNSCC	Cetuximab	II	Tolerable with activity comparable to Cetuximab and platinum combination.	[45]
	R/M HNSCC	Cetuximab	Comparative study	Cetuximab alone showed similar outcomes to Cetuximab with platinum after platinum failure.	[88]
	LA HNSCC	RM-1929 (Cetuximab–IR700 dye conjugate) photoimmunotherapy	I/IIa	It was deemed safe and showed meaningful activity.	NCT02422979 [89]
	OPSCC	Cetuximab combined with Palbociclib	II	The primary endpoint of ORR ≥ 20% was not met.	NCT02101034 [90]
	R/M HNSCC	Cetuximab and Palbociclib vs. Cetuximab alone	II	The addition of Palbociclib to Cetuximab showed no significant improvement in the median OS.	NCT02499120 [91]
	R/M HNSCC	Cetuximab in combination with Palbociclib and Avelumab	I	The combinatorial approach was deemed safe.	NCT03498378 [92]
	R/M HNSCC	Combination of Cetuximab and Ribociclib	I	The MTD was achieved (600 mg daily Ribociclib in combination with a standard dose of Cetuximab for 3 weeks on and 1 week off). Additionally, the combination was deemed safe with clinical activity.	NCT02429089 [93]
	R/M HNSCC	Cetuximab plus Vinorelbine	II	The combination was safe and feasible and showed anticancer activity.	[94]
	Recurrent HNSCC	Palliative weekly Paclitaxel plus Cetuximab		The combinatorial approach was deemed effective leading to a RR of 55% and a median response duration of 5.0 months. Furthermore, the PFS observed was 4.0 months and the OS was 10 months.	[95]
	R/M HNSCC	Weekly Cetuximab with Paclitaxel	Retrospective study	The combinatorial approach was deemed safe and showed antitumoral activity leading to a DCR of 74%, a PFS of 3.9 months, and an OS of 7.6 months.	[96]
	R/M HNSCC	Cetuximab plus Paclitaxel vs. Paclitaxel alone	Retrospective study	Addition of Cetuximab to Paclitaxel after EXTREME leads to moderate benefit and seems to be associated with better PFS and OS.	[97]
	R/M HNSCC	Cetuximab and weekly Paclitaxel	II	Safe and showed clinical activity. An ORR of 54%, 10 (22%) complete responses, a DCR of 80%, a PFS of 4.2 months, and an OS of 8.1 months were observed.	[98]
	R/M HNSCC	Biweekly Cetuximab plus Paclitaxel	II	In previously treated patients (platinum-based and PD-1 immunotherapy) the combination was tolerable and showed promising anticancer activity.	jRCTs051200040 [99]
	R/M HNSCC	Cetuximab plus Paclitaxel	Retrospective study	The combination showed a RR of 37.7%, PFS of 4.5 months, and an OS of 8.9 months, confirming the efficacy and tolerability of the regimen.	[100]
	R/M HNSCC	Weekly Cetuximab and Paclitaxel	Retrospective study	The regimen shows clinical activity and is tolerable.	[101]
	R/M HNSCC	Cetuximab combined with Paclitaxel	Retrospective study	As first-line treatment, it showed an ORR of 43% and a DCR of 79%, while as second-line and later therapies, the ORR achieved was 20% and the DCR was 90%. The PFS and OS were 5.3 and 12.5 months, respectively. Furthermore, it seemed to be more useful after treatment with immune checkpoint inhibitors.	[102]
	R/M HNSCC	Biweekly Cetuximab and Docetaxel	Retrospective study	The treatment was safe and efficient leading to an OS of 8.3 months, PFS of 4.0 months, DCR of 41.9%, and ORR of 12.9%.	[103]
	Stage III/IV HNSCC	Weekly Cetuximab plus Nab-paclitaxel and IMRT	I	A recommended phase II dose was achieved (60 mg/m^2^ of weekly nab-paclitaxel with a standard weekly dose of Cetuximab plus IMRT).	NCT00736619 [104]
	LA NPC	IC with Cetuximab, Carboplatin, and Paclitaxel	Retrospective study	The treatment option was deemed feasible and effective leading to a 3-year recurrence-free survival, locoregional failure-free survival, distant recurrence-free survival, and OS of 75.9%, 79.3%, 84.3%, and 96.3%, respectively.	[105]
	LA HNSCC	IC with Carboplatin, Paclitaxel, and Cetuximab	II	The therapeutic approach was tolerable and effective.	[106]
	LA HNSCC	IC with Cetuximab, Carboplatin, and Nab-paclitaxel followed by standard chemoradiotherapy	II	Showed clinical activity and was deemed safe.	NCT01412229 [107]
	LA HNSCC	IC Cisplatin and Docetaxel with or without Cetuximab	II	The addition of Cetuximab led to higher toxicity and the primary endpoint was not met. Nonetheless, further investigation is warranted.	NCT00623558 [108]
	LA HNSCC	Cetuximab Cisplatin Docetaxel vs. Docetaxel Cisplatin 5-FU	II	Effective and tolerable regimen.	[109]
	R/M NPC	Cetuximab plus Carboplatin and Paclitaxel	Retrospective study	Safe and potentially effective since it led to an ORR of 58.3% and a PFS of 4.1 months.	[110]
	R/M HNSCC	Cetuximab combined with Cisplatin and Docetaxel followed by maintenance with Cetuximab	Retrospective study	It was tolerable and the median PFS (6.9 months) was similar to other studies investigating the same combination.	[111]
	R/M HNSCC	Cetuximab plus Cisplatin with or without Paclitaxel followed by maintenance Cetuximab	II	Addition of Paclitaxel to Cetuximab did not significantly improve PFS or OS.	[112]
	R/M HNSCC	Combination of Cetuximab, Docetaxel, and Cisplatin followed by maintenance Cetuximab	II	Promising activity as a first-line treatment.	NCT01289522 [113]
	R/M HNSCC	Palliative treatment with weekly Cetuximab, Docetaxel, and Cisplatin or Carboplatin	II	The regimen showed promising efficacy and favorable response rates, PFS, and OS when compared to other combinatorial treatments.	NCT01437449 [114]
	R/M HNSCC	Combination of Cetuximab, Paclitaxel, and Carboplatin	II	Acceptable toxicity with promising activity.	[115]
	R/M HNSCC	Cetuximab combined with Carboplatin and Nab-paclitaxel followed by maintenance Nab-paclitaxel and Cetuximab	II	Showed better ORR and OS than historical EXTREME but no improvement in PFS.	NCT02270814 [116]
	R/M HNSCC	Weekly Cetuximab, Paclitaxel, and Carboplatin	Retrospective study	The regimen was deemed safe and showed favorable PFS and OS when compared to the EXTREME regimen. It can be an option for cisplatin-unfit patients.	[117]
	R/M HNSCC	Cetuximab, Paclitaxel, and Carboplatin vs. Cetuximab, 5-FU, and Cisplatin or Carboplatin	II	Cetuximab with Paclitaxel and Carboplatin showed similar efficacy with lower toxicity.	[118]
	R/M HNSCC	Cetuximab plus Cisplatin and Docetaxel followed by maintenance Cetuximab vs. EXTREME regimen (Cetuximab plus Platinum, 5-FU followed by maintenance Cetuximab)	II	The regimen with Cetuximab plus Cisplatin and Docetaxel showed a favorable safety profile compared to the EXTREME regimen, but no significant improvement in OS was observed.	NCT02268695 [119]
	LA HNSCC	TPF IC followed by Cetuximab plus IMRT Carbon ion boost	II	Treatment was tolerable and showed promising results.	NCT01245985 [120,121]
	LA HNSCC	IC with TPF followed by Cetuximab plus Radiotherapy vs. Carboplatin, Fluorouracil, and Radiotherapy	III	No benefits for PFS, OS, and locoregional control were observed for the IC with TPF followed by Cetuximab plus radiotherapy regimen.	NCT01233843 [122]
	Postoperative HNSCC	Cetuximab plus Radiotherapy and Docetaxel or Cisplatin	II	The regimens were feasible and tolerable. The docetaxel combination improved DFS and OS when compared with historical controls.	NCT00084318 [123]
	Resectable OSCC	IC with Cetuximab, Cisplatin, and Paclitaxel followed by weekly Cetuximab plus IMRT	II	The 2-year PFS and OS rates were 80% and 94%, respectively, and the radiation dose reduction led to improved swallowing and nutritional status.	NCT01084083 [124]
	Stage III/IV HNSCC	IC with Cetuximab, Paclitaxel, and Carboplatin followed by Cetuximab plus Carboplatin, Paclitaxel, and Radiotherapy	II	The treatment approach was safe and showed promising survival.	NCT00089297 [125]
	LA HNSCC	IC Cetuximab, Nab-paclitaxel, Cisplatin, and 5-FU followed by Cisplatin plus Radiotherapy	II	High CRR was observed in the primary tumor site.	NCT00736944 [126]
	Stage III/IV larynx/hypopharynx SCC	IC with Docetaxel, Cisplatin, and 5-FU followed by Radiotherapy plus Cisplatin or Cetuximab	II	No treatment approach investigated was superior.	NCT00169247 [127]
	LA HNSCC	IC with Cetuximab, Cisplatin, Paclitaxel, and Everolimus followed by TFHX	I/II	The addition of everolimus to IC showed no benefit.	NCT01133678 [128]
	LA HNSCC	Cetuximab, Docetaxel, and Cisplatin plus Radiation	II	It was impossible to conclude if it was better than TPF with radiation.	[129]
	LA HNSCC	Nab-paclitaxel followed by Cetuximab plus radiotherapy vs Nab-paclitaxel with Cisplatin followed by Cisplatin plus Radiotherapy	II	Due to limited sample size, it was not possible to assess if any regimen was superior.	NCT02573493 [130]
	Stage III/IV OPSCC	IC with Cetuximab, Docetaxel, Cisplatin, and 5-FU	II	The combination dose could not be recommended. However, there were signs of clinical activity and it should be investigated further.	NCT00665392 [131]
	LA HNSCC	IC with Docetaxel, Cisplatin, and 5-FU followed by Cetuximab plus Radiotherapy	II	The regimen was feasible and showed promising results.	NCT0050463 [132]
	Stage III/IV HNSCC	TPF plus Cetuximab followed by weekly Cetuximab and Cisplatin or Carboplatin	II	The regimen led to high toxicity and the study was terminated early.	[133]
	LA HNSCC	IC with Nab-paclitaxel, Cisplatin, and 5-FU with or without Cetuximab	Retrospective comparative study	The addition of Cetuximab to the regimen showed no improvement.	NCT00736944 and NCT01566435 [134]
	LA HNSCC	IC TPF followed by Cetuximab plus Radiotherapy vs. Cisplatin plus Radiotherapy	III	Both tested regimens have similar efficacies and toxicities.	NCT00999700 [135]
	LA HNSCC	IC TPF followed by Cisplatin and Radiotherapy or Cetuximab plus Radiotherapy	II/III	Patients who received IC with TPF showed higher OS, CR, and PFS than the no-IC arm.	NCT01086826 [136]
	Unresectable HNSCC	IC with Cetuximab, Taxotere, Cisplatin, and 5-FU	II	High ORR and CR but with high toxicity.	[137]
	LA HNSCC	IC Cetuximab followed by weekly Cetuximab, Paclitaxel, Carboplatin, and Radiation	II	Led to a 3-year median OS of 4.4 years identical to other trials with paclitaxel-based chemoradiation without Cetuximab	[138]
	LA HNSCC	IC with weekly Cetuximab, Carboplatin, and Paclitaxel followed by Cisplatin plus Radiation	II	Cetuximab combined with Carboplatin and Paclitaxel as IC was deemed feasible with good efficacy.	[139]
	LA HNSCC	Combination of Cetuximab, Nab-paclitaxel, Cisplatin, and Radiation	I/II	Showed a 2-year PFS similar to historical controls.	[140]
	OCSCC	Cetuximab plus TPF followed by Cisplatin and Radiation	II	Shown to be safe and effective.	[141]
	Postoperative III–IVb HNSCC	Weekly Cetuximab, Docetaxel, and Radiotherapy		Deemed feasible and safe and led to 1- and 2-year DFS rates of 91 and 55%, respectively.	[142]
	LA HNSCC	IC with Cetuximab, Cisplatin, and Docetaxel followed by concurrent Radiotherapy with Cisplatin and Cetuximab followed by maintenance Cetuximab	II	Led to great long-term survival with 3-year PFS and OS rates of 70% and 74%.	[143]
	Laryngeal cancer	IC with Docetaxel, Cisplatin, and 5-FU followed by Carboplatin or Cetuximab plus Radiotherapy		Cetuximab is a reasonable alternative, but no significant improvement was observed.	[144]
	LA HNSCC	IC with Cisplatin, Docetaxel, and Capecitabine followed by Cetuximab and Radiotherapy	I/II	Discontinued early due to high toxicity.	[145]
	LA HNSCC	IC with Docetaxel, Cisplatin, 5-FU, and Cetuximab followed by Cetuximab and Radiotherapy	Retrospective study	Deemed safe with promising efficacy.	[146]
	LA HNSCC	IC with Cetuximab, Paclitaxel, and Carboplatin followed by Cetuximab, 5-FU, Hydroxyurea, and Radiotherapy or Cisplatin, Cetuximab, and Radiotherapy	II	Tolerable and led to long-term control of LA HNSCC.	[147]
Erlotinib, EGFR TK Inhibitor	Oral premalignant lesions	Erlotinib		Did not improve CFS.	NCT00402779 [148]
	HNC	Erlotinib	Window-of-opportunity study	The results suggest that early in-treatment PET/CT can predict patients’ response to erlotinib.	NCT00601913 [149]
	R/M HNSCC	Erlotinib	II	Erlotinib was deemed safe and led to prolonged disease stabilization.	[70]
	LA HNSCC	Erlotinib	Pilot study	Well tolerated. Nine patients showed tumor reduction at the time of surgery.	[150]
	R/M HNSCC	Erlotinib	II	Long-term erlotinib, although safe, showed low tolerability. Nonetheless, a potential improvement in survival when compared to historical controls was observed.	[151]
	LA HNC	Erlotinib plus Docetaxel	I	High toxicity observed. However, the dose for phase II trials was determined (Docetaxel 35 mg/m^2^ and Erlotinib 50 mg).	NCT00049283 [152]
	HNC	Erlotinib combined with Docetaxel and IMRT	II	The regimen was deemed safe and should be investigated further in selected patients. May be useful for patients who cannot receive cisplatin treatment.	NCT00720304 [153]
	R/M HNSCC	Erlotinib plus Cisplatin and Docetaxel	II	The combination led to an ORR of 62%, a median PFS of 6.1 months, and an OS of 11.0 months which compares favorably with historical controls of treatment with Cisplatin and Docetaxel.	NCT00076310 [154]
	LA HNSCC	IC with Paclitaxel, Carboplatin, 5-FU, and Bevacizumab followed by Radiotherapy, Erlotinib, Paclitaxel, and Bevacizumab	II	High efficacy with manageable toxicity. The estimated PFS and OS rates were 71% and 82%, respectively.	[155]
Lapatinib, EGFR and HER2 TK Inhibitor	LA HNSCC	Lapatinib	II	Short-term administration of Lapatinib showed clinical activity.	NCT00371566 [72]
	Malignant tumors of the salivary glands	Lapatinib ditosylate	II	Lapatinib administration was shown to be tolerable, leading, in 36% of patients, to a 6-month tumor stabilization; however, the effects observed were mainly cytostatic.	NCT00095563 [156]
	R/M HNSCC	Lapatinib	II	Well tolerated but showed no clinical activity.	[157]
	HNSCC	IC with Lapatinib, Carboplatin, and Paclitaxel followed by Transoral surgery and neck dissection	II	The therapeutic approach was feasible and led to DSS, PFS, and OS rates of 100%, 97%, and 97%, respectively, with a median follow-up of 4.9 years.	NCT01612351 [158]
Dacomitinib, EGFR, HER2, and HER4 TK Inhibitor	LA HNSCC	Dacomitinib		Administration of Dacomitinib with a gastrostomy feeding tube led to reduced AUC and Cmax compared to oral dosing of intact immediate-release tablets.	NCT01484847 [159]
	R/M HNSCC	Dacomitinib	II	Manageable toxicity with clinical activity. Primary endpoint was met.	[80]
	R/M HNSCC	Dacomitinib	II	The treatment was tolerable, showing clinical efficacy in platinum-refractory R/M HNSCC patients.	NCT01449201 [160]
Poziotinib, EGFR, HER2, and HER4 TK Inhibitor	R/M HNSCC	Poziotinib	II	Poziotinib led to an ORR of 22.4%, median PFS of 4.0 months, and median OS of 7.6 months.	NCT02216916 [161]
Panitumumab, EGFR Monoclonal Antibody	Advanced HNSCC	Panitumumab	II	Showed moderate clinical activity with a considerable disease-control rate. It was deemed safe.	NCT02643056 [162]
	R/M HNSCC	Panitumumab	II	Showed limited activity, leading to ORR of 4%, DCR of 39%, PFS of 1.4 months, and OS of 5.1 months.	NCT00446446 [163]
	R/M HNSCC	Panitumumab plus Paclitaxel	II	Showed clinical activity with a good safety profile.	NCT01264328 [164]
	LA HNSCC	IC with Panitumumab and Paclitaxel followed by Panitumumab plus Radiotherapy	II	Worse toxicity than anticipated but showed clinical activity.	[165]
	LA HNSCC	Panitumumab plus Paclitaxel, Carboplatin, and IMRT	I	Safe with good activity, with 95% of patients remaining disease-free at median follow-up of 21 months.	[166]
	R/M HNSCC	Panitumumab, Docetaxel, and Cisplatin vs. Docetaxel plus Cisplatin	II	Addition of Panitumumab to Docetaxel plus Cisplatin increased the PFS (6.9 vs 5.5 months) and ORR (44% vs 37%). However, a worse OS was observed (12.9 vs 13.8 months).	NCT00454779 [167]
Nimotuzumab, EGFR Monoclonal Antibody	Resistant LA NPC	Nimotuzumab	II	Showed clinical activity with mild toxicity.	NCT04508816 [168]
Duligotuzumab EGFR and HER3 Monoclonal Antibody	R/M HNSCC	Duligotuzumab vs. Cetuximab	II	Duligotuzumab did not improve the outcomes when compared with Cetuximab.	NCT01577173 [169]
	R/M HNSCC	Duligotuzumab plus Cisplatin and 5-FU or Carboplatin and Paclitaxel	Ib	Both combinations showed good activity but with increased toxicity.	NCT01911598 [170]
A166, HER2 Antibody Drug Conjugate	Solid tumors (including SGC)	A166	I/II	In phase I, the recommended concentration was obtained. Manageable toxicity and promising clinical activity were observed.	NCT03602079 [171]
Gefitinib, EGFR TK Inhibitor	Solid tumors (including HNSCC)	Gefitinib	I	Showed activity and a manageable safety profile.	[172]
	Cutaneous HNSCC	Gefitinib	II	Showed clinical activity and was deemed safe.	[173]
	R/M NPC	Gefitinib	II	Weak response rate but well tolerated.	[174]
	SGC	Gefitinib	II	No significant clinical activity was observed.	NCT00509002 [175]
	R/M HNSCC	Gefitinib	II	Was well tolerated. An ORR of 10.6%, a DCR of 53%, a PFS of 3.4 months, and an OS of 8.1 months were observed.	[176]
	R/M HNSCC	Gefitinib	II	Gefitinib monotherapy showed clinical activity.	[177]
	R/M HNSCC	Gefitinib	II	Gefitinib showed less activity at 250 mg than at 500 mg.	[178]
	Advanced solid tumors (including HNC)	Gefitinib plus Docetaxel	I	A recommended dose for phase II trials was achieved (gefitinib 2250 mg on days 1 and 2, followed by docetaxel 75 mg/m^2^ on day 3).	[179]
	R/M HNSCC	Gefitinib plus Docetaxel vs. Docetaxel alone	III	The combination showed good tolerability but no outcome improvements.	NCT00088907 [180]
	LA HNSCC	Gefitinib plus Paclitaxel and Radiotherapy	I	Addition of Gefitinib to Paclitaxel with radiotherapy does not seem to improve response rates.	[181]
	LA HNC	Carboplatin or Paclitaxel IC followed by 5-FU, Hydroxyurea, Radiotherapy, and Gefitinib followed by continued Gefitinib	II	The treatment approach was feasible and showed promising activity.	[182]
Zalutumumab, EGFR Monoclonal Antibody	Advanced HNSCC	Zalutumumab	I/II	Preliminary data indicated promising tumor response and the MTD was achieved.	[183]
	Platinum-refractory HNSCC	Zalutumumab	II	Zalutumumab led to an ORR of 5.7%, a DCR of 39.8%, an OS of 5.3 months, and a PFS of 2.1 months.	NCT00542308 [184]
	R/M HNSCC	Zalutumumab	III	In patients who failed platinum-based chemotherapy, it improved the PFS, but no improvement was observed in OS.	NCT00382031 [185]
CDX-3379, HER3 Monoclonal Antibody	HNSCC	CDX-3379	Window-of- opportunity	The regimen was tolerable and led to tumor regression.	NCT02473731 [186]
Barecetamab, HER3 Monoclonal Antibody	R/M HNSCC	Barecetamab vs. Barecetamab plus Cetuximab	Ia/Ib	The recommended dose for phase II trials was achieved. Both treatment approaches were deemed safe and led to meaningful anticancer activity.	NCT03552406 [187]
Afatinib, EGFR, HER2, and HER4 TK Inhibitor	HNSCC	Afatinib	II	Afatinib induced a high rate of metabolic response. Patients who responded to treatment had a higher expression of pERK1/2 and lower expressions of pHER4 and pRB1.	NCT01415674 [188]
	HNSCC	Afatinib	II	Afatinib led to a high rate of FDG-PET partial metabolic response and partial response. Furthermore, it was deemed safe to be administered before surgery	NCT01538381 [189]
	HNSCC	Afatinib	III	DFS showed no improvement after treatment with afatinib after CRT while increasing the number of adverse events.	NCT01345669 [190,191]
	R/M HNSCC	Afatinib vs. Methotrexate as second-line treatment	III	Afatinib was deemed safe and improved PFS.	NCT01345682 [77]
	R/M HNSCC	Afatinib as a second-line treatment	III	Effective by oral and tube administration with manageable toxicity.	NCT01345682 [192]
	R/M HNSCC	Afatinib vs. Methotrexate as a second-line treatment	III	Afatinib was deemed safe and improved PFS.	NCT01856478 [193]
	R/M HNSCC	Afatinib as second line treatment	III	Effective by oral and tube administration with manageable toxicity.	NCT01345682 [192]
	R/M HNSCC	Afatinib vs. Cetuximab	II	Afatinib showed similar efficacy to Cetuximab but with higher toxicity.	[76]
	OSCC	Afatinib plus Ribavirin and weekly Carboplatin and Paclitaxel	I	The combinatorial approach was safe and well tolerated.	[194]
	LA HNSCC	Afatinib, Carboplatin, and Paclitaxel as IC followed by Cisplatin and Radiotherapy	I	The MTD was achieved.	NCT01732640 [195]

Abbreviations: 5-FU: 5-fluorouracil; AUC: area under the concentration–time curve; CFS: cancer-free survival; C_max_: maximum observed concentration; CR: complete response; CRR: complete response rate; CRT: chemoradiation; DCR: disease-control rate; DFS: disease-free survival; DSS: disease-specific survival; EGFR: epidermal growth factor receptor; FDG-PET: 18-Fluoro-deoxyglucose positron emission tomography; HER3: human epidermal growth factor receptor 3; HNC: head and neck cancer; HNSCC: head and neck squamous cell carcinoma; IC: induction chemotherapy; IMRT: intensity-modulated radiation therapy; LA HNC: locoregionally advanced head and neck cancer; LA HNSCC: locoregionally advanced head and neck squamous cell carcinoma; LA NPC: locoregionally advanced nasopharyngeal carcinoma; MTD: maximum tolerated dose; Nab-paclitaxel: nanoparticle albumin-bound paclitaxel; NPC: nasopharyngeal carcinoma; OCSCC: oral cavity squamous cell carcinoma; OPSCC: oropharyngeal squamous cell carcinoma; ORR: objective response rate; OS: overall survival; OSCC: oral squamous cell carcinoma; PD-1: programmed cell death 1; PET/CT: positron emission tomography/computed tomography; PFS: progression-free survival; R/M HNSCC: recurrent or metastatic head and neck squamous cell carcinoma; R/M NPC: recurrent or metastatic nasopharyngeal carcinoma; R/M SGC: recurrent or metastatic salivary gland carcinoma; RR: response rate; SGC: salivary gland cancer; TFHX: paclitaxel, fluorouracil, hydroxyurea, and radiotherapy; TK: tyrosine kinase; TPF: docetaxel, cisplatin, and 5-FU.

## 3. Overview of Classic Microtubule-Targeting Agents (MTAs) versus Second-Generation of Antimitotics (SGAs)

The deregulation of cellular metabolism and the alterations in the cell cycle of tumor cells has triggered vast research on therapies focusing on antimitotic drugs, mostly MTAs, which can be categorized into two groups based on their distinct mechanisms of action: microtubule destabilizers, such as the vinca alkaloids, which impede the polymerization of microtubules, and microtubule stabilizers, such as taxanes, which enhance microtubule polymerization [196]. Both classes exert their effects by disrupting the proper formation of the mitotic spindle, leading to the activation of the SAC, subsequent arrest of the cell cycle at the mitotic phase, and typically followed by cell death [197]. However, prolonged mitotic arrest caused by MTAs can trigger the exit of cells from mitosis without undergoing cytokinesis, resulting in the generation of tetraploid cells. This process, known as mitotic slippage, arises from the gradual and continuous degradation of cyclin B1, even in the presence of an active SAC [198]. Cells that have undergone mitotic slippage have three possible outcomes: entering a senescent state, undergoing post-mitotic death, or continuing to divide. Therefore, mitotic slippage, in conjunction with factors such as efflux pumps, mutations in tubulin genes, and compromised apoptotic signaling, significantly contribute to the therapeutic inefficacy of MTAs [199]. Moreover, treatment with MTAs frequently gives rise to neurological and myeloid toxicity [200]. Because of all these limitations, the need arose to develop drugs targeting mitotic proteins, especially kinases and motor proteins. Thus, second-generation antimitotics (SGAs) emerged with inhibitors of MPS1, polo-like kinase 1 (PLK1), Aurora kinases, KSP, and centromeric protein E (CENP-E) [201]. From the perspective of SAC response, the small molecule inhibitors known as SGAs can be classified into two distinct groups [201]. The first group comprises mitotic blockers, which target essential proteins such as CENP-E, KSP, and PLK1. These inhibitors trigger the activation of the SAC, leading to a prolonged delay in mitosis with the ultimate expectation of inducing cell death. Conversely, the second group, known as mitotic drivers, includes inhibitors targeting MPS1 and Aurora B kinase. These compounds override the SAC, causing premature exit from mitosis, accompanied by extensive chromosome mis-segregation. Consequently, this results in the emergence of chromosome aberrations that render daughter cells non-viable. Given this phenotype, the combination of MPS1 inhibition with MTA co-treatment has been explored in different cancer types (Table 2).

## 4. Targeting Monopolar Spindle 1 (MPS1) in Cancer

**Figure 2:**
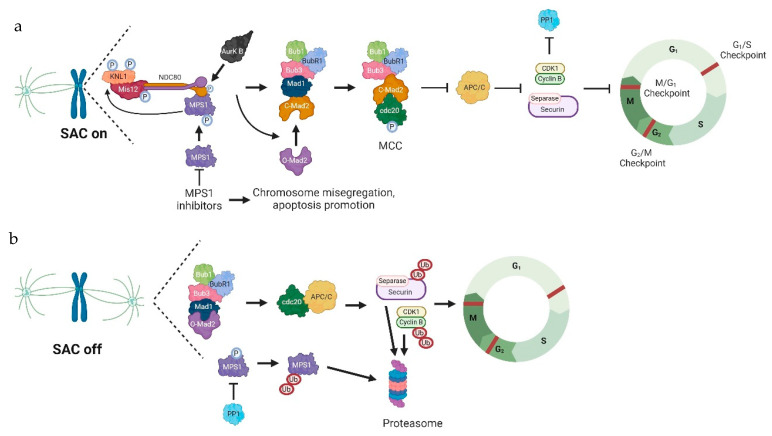
MPS1 role in SAC function. (**a**) Unattached kinetochores activate the SAC. Aurora B promotes the recruitment of activated/phosphorylated MPS1 to the kinetochore by phosphorylating Hec1 in the NDC80 complex. MPS1 then binds the NDC80 complex and phosphorylates KNL1, which is essential for the recruitment of SAC proteins. MPS1 also plays a role in the conversion of O-Mad2 to C-Mad2, leading to MCC formation. Cdc20 binds to the MCC instead of the APC/C, inhibiting the transition from the M phase to the G1 phase of the cell cycle. MPS1 inhibitors will suppress SAC activation leading to chromosome mis-segregation, increasing cell death signaling. (**b**) Attached kinetochores lead to SAC inactivation. With SAC inactive, Cdc20 binds to the APC/C, which leads to cyclin B and securin ubiquitination and degradation. PP1 is, thus, active and inhibits MPS1 function. Consequently, the cell cycle can progress to the G1 phase. Abbreviations: APC/C, anaphase-promoting complex/cyclosome; AurK B, aurora kinase B; BUB1, budding uninhibited by benzimidazoles 1; BUB3, budding uninhibited by benzimidazoles 3; BUBR1, budding uninhibited by benzimidazole-related 1; C-Mad2, closed mitotic arrest deficiency 2; Cdc20, cell division cycle 20 homolog; CDK1, cyclin-dependent kinase; KNL1, kinetochore null protein 1 or kinetochore scaffold 1; Mad1, mitotic arrest deficiency 1; MCC, mitotic checkpoint complex; MPS1, monopolar spindle 1; O-Mad2, open mitotic arrest deficiency 2; SAC, spindle assembly checkpoint.

MPS1 is a dual-specific protein kinase composed of 857 residues that phosphorylates serine and threonine residues [203]. It is located on chromosome 6q13-q21 and functions as a crucial regulator of the SAC, playing a pivotal role in controlling cell cycle progression and preserving genomic stability (Figure 2) [204]. In addition, it is involved in the regulation of cytochrome c release, response to DNA damage, and centrosome duplication [205]. MPS1 is a vital regulator of chromosome alignment during metaphase, ensuring that aneuploidy is prevented. SAC is responsible for monitoring chromosome segregation and halting cell division upon detecting any anomalies. MPS1 is recruited to unattached kinetochores where it is suggested it binds to the NDC80 complex, part of the KMN network [206]. There, MPS1 phosphorylates kinetochore scaffold 1, another component of the KMN network that is essential for the recruitment of SAC proteins such as budding uninhibited by benzimidazoles (BUB) 3, which is bound to BUB1. MPS1 also phosphorylates BUB1, promoting the recruitment of the mitotic arrest deficient (MAD) 1:C–MAD2 complex [206,207]. The activity of MPS1 is essential for the formation of the mitotic checkpoint complex (MCC) and activation of the SAC [208]. MCC inhibits the anaphase-promoting complex/Cyclosome (APC/C), thus preventing premature initiation of anaphase. The interaction between MPS1 and APC is critical for cell cycle regulation. Specifically, when APC/C binds to its positive regulator, cell-division cycle protein 20 (CDC20), SAC activity is inhibited. Conversely, SAC can interfere with APC/C-CDC20 binding through MAD2, a negative regulator of APC/C. MPS1 is involved in this process through the phosphorylation of Mad1, which promotes the interaction of MAD2 with CDC20 [206]. In this negative feedback loop, if chromosome misalignment occurs, activated SAC can result in APC/C inactivation, leading to MPS1 stabilization and enhanced checkpoint activity. As chromosomes become aligned on the equatorial plate, SAC activity gradually reduces, and APC/C is activated, ultimately resulting in the degradation of MPS1. This process reduces checkpoint activity and initiates the next cell cycle [209,210]. Another level of MPS1 activity regulation comes from protein phosphatase 1 (PP1). PP1 is a serine/threonine phosphatase involved in essential processes such as the cell cycle, apoptosis, etc. [211]. This protein directly prevents MPS1 T-loop autophosphorylation, leading to the inactivation of this kinase at metaphase kinetochores and in the cytoplasm. This inactivation is crucial for the rapid silencing of SAC and timely progression through mitosis [212]. 

In addition to SAC overdriving, inhibition of MPS1 leads to chromosome mis-segregation and consequently to cell death. Nonetheless, in aneuploid cancer cells, it was shown that SAC inactivation is not necessary for MPS1 inhibition to cause cell death since MPS1 is also involved in the regulation of cytochrome c release [213]. Therefore, the relevancy of MPS1 for tumor cell viability has led to the development of compounds for this therapeutic target over the last few years. 

Currently, there are six MPS1 inhibitors approved and in use for clinical trials; these are S81694, BOS172722, BAY 1217389, BAY 1161909, CFI-402257, and BAL0891 [18,34,35,36,37,38,39,40,41,42,43] (Table 1). 

In human colorectal carcinoma cell lines, BOS172722 led to growth inhibition while early mitotic exit and induction of aneuploidy were observed in triple-negative breast cancer cells (TNBCs). Synergistic effects in combination with paclitaxel were also observed both in vitro and in vivo [214]. An ongoing phase 1/1b clinical trial is assessing the safety and maximum tolerated dose of BOS172722 in combination with Paclitaxel in advanced nonhematologic malignancies (NCT03328494) [215].

S81694 leads to mitotic overriding, chromosomal alignment errors, induction of apoptosis, and consequently cell death, and showed antitumor effects both in vitro and in vivo [216]. A phase I clinical trial with patients with advanced metastatic solid tumors showed that administration of S81694 was safe, with an 8.1-week median progression-free survival (PFS). Nonetheless, the recommended phase II dose was not defined since combination with paclitaxel in breast cancer patients was prioritized due to promising preclinical results [216]. However, the study was terminated due to disappointing clinical activity.

BAY 1161909 in an osteosarcoma cell line (U-2 OS) induced aneuploidy, while in a cisplatin-resistant ovarian tumor xenograft model (A2780cis), moderate efficacy was observed with a good safety profile and improved inhibition of tumor growth when compared with cisplatin. BAY 1217389 showed similar results in the same murine model [217]. In addition, both inhibitors reported moderate efficacy as monotherapy in several tumor murine models mainly because of tolerability issues [218]. Nonetheless, in TNBC xenografts, antitumor synergistic effects were observed for these inhibitors when in combination with paclitaxel [218]. In accordance, tumor growth delay was observed with the combination of BAY 1217389 and paclitaxel while the combination of paclitaxel and BAY 1161909 led to full tumor remission. In non-small cell lung cancer (NSCLC) xenograft models, BAY 1217389 and paclitaxel showed better results than paclitaxel alone. Moreover, paclitaxel in combination with BAY 1161909 showed good tolerability and reduced tumor growth and size [217]. Based on the encouraging outcomes observed in preclinical in vivo studies, demonstrating a synergistic effect between MPS1 inhibitors and taxanes, several small molecules have undergone clinical trials in combination with paclitaxel. In a clinical trial (NCT02138812) evaluating the efficacy of BAY1161909 in the treatment of advanced solid malignancies, the combination of BAY1161909 and paclitaxel at doses of 75 mg/m^2^ and 90 mg/m^2^ yielded five (14%) and four (14%) partial responses (PRs), respectively [28]. Despite these promising results, Bayer suspended the clinical trial and replaced BAY 1161909 with BAY 1217389 (NCT02366949). The results of this phase I clinical trial for patients with advanced malignancies showed that the combination of paclitaxel and BAY 1217389 led to high toxicity, with bone marrow toxicity as a significant adverse reaction. Nonetheless, a maximum tolerated dose was achieved for the combination [202].

CFI-402257 is a potent inhibitor of MPS1 that leads to errors in chromosome segregation, DNA damage, and consequently cell death [219]. In lung cancer, both in vitro and in vivo, this inhibitor showed promising antitumor effects by enhancing apoptosis and polyploidy [220]. The administration of CFI-402257 in hepatocellular carcinoma increased apoptosis and aneuploidy and suppressed tumor growth while leading to the activation of a DNA pathway involved in senescence-associated secretory phenotype. In addition, in a murine model, combining an anti-PD-1 drug with CFI-402257 increased survival [221]. Furthermore, the addition of CFI-402257 to breast cancer cell lines resistant to the inhibition of CDK4/6 demonstrated improved anticancer effects. Thus, it is suggested that CFI-402257 administration could be beneficial to ER^+^ breast cancer patients resistant to CDK4/6-targeting therapies [220]. In malignant mesothelioma cell lines, CFI-402257 led to increased aneuploidy and cell death while it led to tumor growth delay in vivo. Additionally, when combined with cisplatin and pemetrexed, it showed increased anticancer effects [222]. Nonetheless, it is important to note that determining the long-term tolerance and toxicity associated with the use of MPS1 inhibitors requires further investigation [28,52]. All the completed or ongoing clinical trials investigating MPS1 inhibitors have been performed on patients with advanced malignancies (solid tumors) or breast cancer, with no reference to treatments in patients with oral or head and neck cancer.

## 5. Clinical Relevance of MPS1 and EGFR Co-Targeting for Head and Neck Cancer

Co-targeting MPS1 and EGFR could be a promising therapeutic strategy for the treatment of head and neck cancer, particularly OSCC. On one hand, MPS1 is a key component of the spindle assembly checkpoint, ensuring proper chromosome segregation during mitosis, and its overexpression is correlated with poor prognosis in several cancers. On the other hand, EGFR, a receptor tyrosine kinase, is overexpressed in a significant majority of head and neck cancers and is associated with aggressive tumor behavior and resistance to conventional therapies. Combining inhibitors of MPS1 with EGFR-targeted therapies such as Cetuximab could synergistically enhance treatment efficacy. By simultaneously disrupting mitotic control and EGFR signaling, this approach may effectively halt tumor progression, reduce resistance to treatment, and improve overall survival rates. Preclinical studies have shown that dual inhibition can lead to increased apoptosis and decreased proliferation of cancer cells compared to targeting either pathway alone. Furthermore, this combinatorial strategy aligns with precision medicine goals, offering tailored treatment plans based on the molecular profile of the tumor.

Thus, to explore a possible co-targeted therapy of both EGFR and MPS1 for the treatment of HNSCC, we used the UALCAN database to obtain data mainly concerning MPS1 expression and head and neck cancer. UALCAN is a comprehensive computational platform that employs advanced bioinformatics techniques and data analytics to facilitate integrative analysis and visualization of cancer genomics data. It serves as a valuable resource for researchers and clinicians, offering access to a vast array of publicly available cancer transcriptomics and clinical data [223]. UALCAN uses The Cancer Genome Atlas (TCGA) database to display transcriptomic results, which are presented in transcripts per million. For proteomic information, the Clinical Proteomic Tumor Analysis Consortium (CPTAC) database is used and shown in Z-values. Z-values represent standard deviations from the median across HNSCC samples. Pearson correlation analysis is performed by UALCAN for correlation analysis. For transcriptomic data, the results were provided by UALCAN as transcripts per million, while for proteomic information, the results were expressed as Z-values, which represent standard deviations from the median across HNSCC samples. For correlation analysis, UALCAN used Pearson correlation analysis and provided the Pearson correlation coefficient values. For survival plots, Kaplan–Meyer curves were generated to express HNSCC patient survival according to MPS1 expression and clinicopathological features. Data are shown as the mean ± standard deviation (SD). Statistical significance represented as *p* values were provided by UALCAN. Z-values represent standard deviations from the median across HNSCC samples. Log2 Spectral count ratio values from CPTAC were first normalized within each sample profile and then normalized across samples.

**Figure 3:**
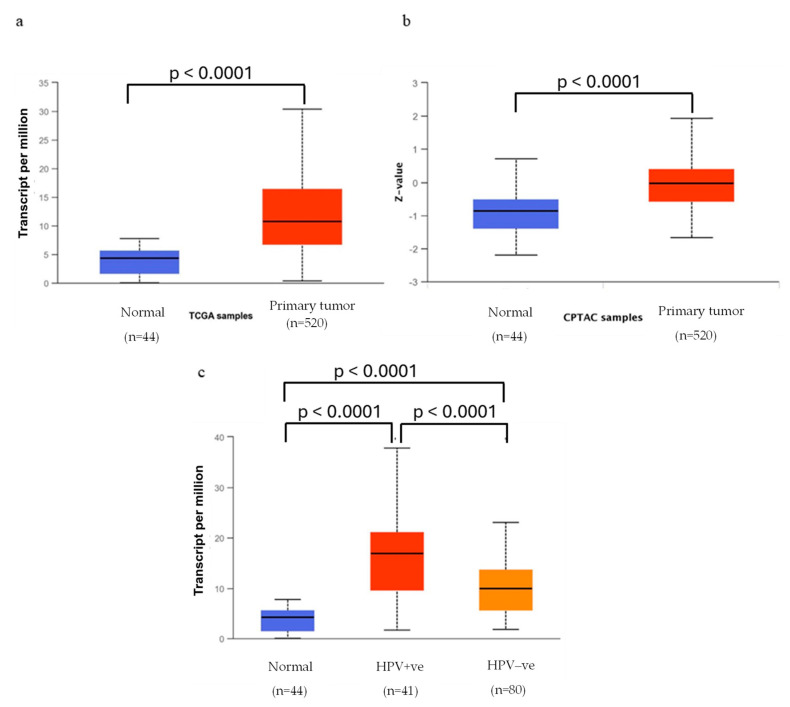
Transcriptional (**a**) and proteomic (**b**) expression of MPS1 in head and neck squamous carcinoma based on sample types (normal and primary tumors). (**c**) MPS1 mRNA expression levels by tumor samples’ HPV status. TCGA = The Cancer Genome Atlas; CPTAC = Clinical Proteomic Tumor Analysis Consortium.

The analysis performed with the UALCAN web resource showed that both MPS1 mRNA and protein expression levels are upregulated in HNSCC primary tumors, with a median value of transcripts per million approximately 2.5 times higher for tumor samples (Figure 3a,b). In HNSCC samples with lymphovascular invasion, the MPS1 gene expression was shown to be upregulated when compared to normal samples, and samples with high expression of MPS1 were associated with worse prognosis [224], highlighting the potential of MPS1 targeting for head and neck cancer. Moreover, overexpression of MPS1 has been associated with shorter periods of recurrence and survival time and is correlated with poor prognosis in several types of cancers [205,225]. 

Most HPV-positive HNSCC tumors originate from the oropharynx where there is a correlation between HPV status and patient survival since HPV-positive tumors are usually more responsive to treatment and associated with favorable survival [226,227]. The UALCAN analysis of MPS1 transcriptional expression by HPV status showed a statistically significant increase in MPS1 expression levels in both HPV-positive and negative samples when compared to normal samples. In addition, a significant difference was observed when comparing both HPV statuses, suggesting that HPV-positive HNSCC has a higher expression of MPS1 (Figure 3c). 

**Figure 4:**
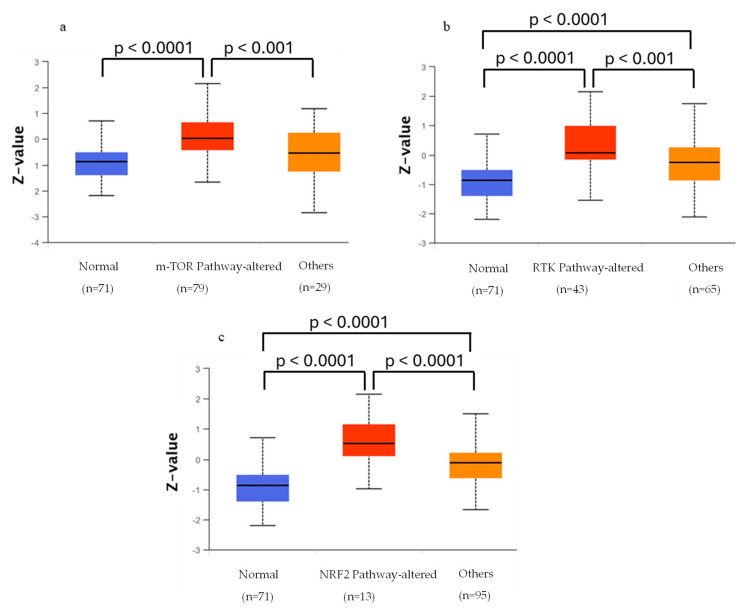
Correlation analysis of MPS1 overexpression and altered (**a**) mTOR, (**b**) RTK, and (**c**) NRF2 pathways.

According to UALCAN data, increased levels of MPS1 are linked with the presence of altered mTOR, RTK, and nuclear factor erythroid 2 related factor 2 (NRF2) pathways (Figure 4).

The NRF2 pathway is involved in the regulation of oxidative stress response and was recently shown to be frequently mutated in HPV-negative HNSCC but rarely so in HPV-positive HNSCC [228]. In addition, high levels of NRF2 are associated with poor prognosis and thus since this pathway acts as a defensive mechanism, its alteration could explain in part why HPV-negative HNSCC is more unresponsive to treatment [229]. NRF2 is a transcription factor involved in the expression of antioxidant proteins and chaperones, among others. Its function is negatively regulated by KEAP1, a BTB-Kelch protein that is part of the E3 ubiquitin kinase complex, which is inactivated during oxidative stress [228,230]. In estrogen-induced breast cancer, NRF2 was shown to prevent oxidative DNA damage by upregulating 8-Oxoguanine DNA glycosylase, a DNA repair protein [231]. MPS1 also plays a role in the repair of oxidative DNA lesions by phosphorylating MDM2, which leads to the loosening of the chromatin, allowing DNA cleavage by DNA repair proteins [232]. In cancer cells, reactive oxygen species are associated with the promotion of tumorigenesis and chemoresistance and can increase tumor angiogenesis [233]. In this sense, co-inhibition of MPS1 and NRF2 could lead to increased cell death through oxidative stress and thus the exploration of this combination could be of interest mainly for the treatment of HPV-negative HNSCC.

**Figure 5:**
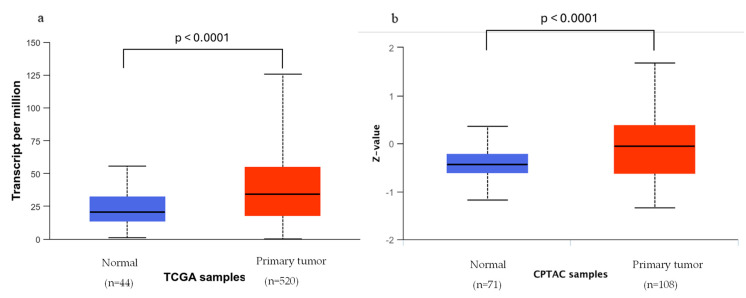
Transcriptional (**a**) and protein (**b**) expression of EGFR in normal and primary tumor samples.

In several types of cancers, MPS1 was shown to be associated with the AKT/mTOR pathway [24,234,235,236,237,238]. This pathway is involved in tumor cell proliferation and survival and is reported to be overexpressed in 90% of HNSCC tumors [239]. Moreover, it has been shown that increased MPS1 protein expression leads to an increase in phosphorylated AKT proteins [234,235]. However, it is not clear if MPS1 directly phosphorylates AKT or if other proteins are involved in this phosphorylation. Nonetheless, in both esophageal squamous cell carcinoma (ESCC) and hepatocellular carcinoma cells, MPS1 activates the AKT/mTOR pathway, promoting cell proliferation and migration [24,238]. Furthermore, in ESCC, this activation was shown to be regulated by Annexin A2 [238], while in ovarian cancer and renal cancer, it was shown that MPS1 inhibits apoptosis through this pathway [235]. Additionally, EGFR, an RTK, was shown to activate the PI3K/AKT/mTOR pathway, and it is overexpressed in HNSCC, as corroborated by the UALCAN analysis (Figure 5) [240].

Nonetheless, alterations in this pathway can lead to resistance to EGFR inhibition [241]. Recently, in lung cancer cells, inhibition of Aurora B, a kinase involved in chromosomal segregation and cytokinesis, was shown to prevent and overcome EGFR inhibition resistance by increasing apoptosis mediated by BIM and PUMA, BH3 domain-containing proteins that are involved in the activation of proapoptotic proteins [242]. Aurora B also plays a role in the recruitment of MPS1 to unattached kinetochores by phosphorylating Hec1, a NDC80 complex protein [243]. This complex is involved in the attachment of microtubules to chromosomes [244]. In addition, it was also reported that MPS1 is essential for activating regulators of Aurora B activity and consequently for promoting Aurora B recruitment to centromeres [245,246]. Thus, since MPS1 has a role in both the AKT/mTOR pathway and in the activation of Aurora B activity, we believe that a combinatorial approach targeting EGFR and MPS1 could lead to an enhanced antiproliferative effect and increased cell death, and also prevent or overcome resistance to EGFR inhibition therapy in HNSCC. 

## 6. Conclusions and Future Perspectives 

Currently, no studies have explored the combination of MPS1 and EGFR-targeting in any cancer type, nor have there been studies into the use of EGFR with other second-generation antimitotic agents in HNSCC. Thus, this constitutes a novel approach to be evaluated in both in vitro and in vivo models that could potentially enhance EGFR-targeting drug efficacy and/or overcome cancer resistance to anti-EGFR drugs.

Additionally, the potential benefits of co-inhibiting MPS1 and NRF2, particularly for HPV-negative HNSCC, warrant further investigation. Co-inhibiting MPS1 and NRF2 could induce oxidative stress and enhance cell death in cancer cells. Investigating this combination could provide insights into new therapeutic approaches for treating HPV-negative HNSCC, a subtype often associated with worse outcomes compared to HPV-positive HNSCC.

Research should also focus on the interplay between MPS1, the AKT/mTOR pathway, and Aurora B kinase activity. The AKT/mTOR pathway is a critical regulator of cell proliferation, survival, and metabolism, and is overexpressed in a significant majority of HNSCC tumors. MPS1 has been shown to interact with this pathway, promoting cell proliferation and migration. Additionally, Aurora B kinase is involved in chromosomal segregation and cytokinesis, and its inhibition has been demonstrated to overcome resistance to EGFR inhibition in various cancers. By targeting both MPS1 and EGFR, along with modulating the AKT/mTOR and Aurora B pathways, we could potentially develop a multi-faceted approach to overcome resistance mechanisms in HNSCC. For example, combining MPS1 and Aurora B inhibitors with EGFR-targeted therapies could disrupt multiple critical points in the cell cycle and signaling pathways, leading to enhanced apoptosis and reduced proliferation of cancer cells. 

By addressing these research gaps, we can move closer to developing more effective, targeted treatments for HNC, ultimately improving patient outcomes and survival rates. Integrating co-targeting strategies and exploring the interplay between key signaling pathways will provide a holistic approach to HNC treatment. Continued research and clinical trials will be essential to translate these findings into viable therapeutic options, paving the way for personalized and precision medicine in cancer treatment.

## Figures and Tables

**Table 2:** Clinical trials targeting MPS1 alone or in combination for cancer treatment.

Drug Name	Disease	Intervention	Phase	Results	NCT Identifier	References
BAY-1217389 (Completed)	Advanced malignancies (solid tumors)	BAY-1217389 + paclitaxel	I	The combination of BAY with paclitaxel was associated with considerable toxicity without a therapeutic window.	NCT02366949	[202]
BAY-1161909 (Terminated)	Advanced malignancies (solid tumors)	BAY-1161909 + paclitaxel	I	As another MPS-1 inhibitor was being developed in parallel (BAY-1217389), the strategic decision was to move forward with the development of the follow-up compound only.	NCT02138812	
BOS-172722 (Completed)	Advanced Nonhematologic Malignancies	BOS-172722 + paclitaxel	I	Without results.	NCT03328494	
BAL0891 (Recruiting)	Advanced Solid Tumors	BAL0891 monotherapy and BAL0891 + carboplatin or paclitaxel	I	Without results.	NCT05768932	
CFI-402257 (Active, not recruiting)	Advanced Solid Cancers, Breast Cancer	CFI-402257 + Fulvestrant	I	Without results, Active, Estimated study completion date: May 2027	NCT02792465	
	Breast Cancer	CFI-402257 + paclitaxel	II	The phase 2 recommended doses were CFI-402257 168 mg + Paclitaxel 80 mg/m^2^. The overall response rate was 5.9% and the clinical benefit rate was 47.1%.	NCT03568422	
CFI-402257 (Recruiting)	Advanced malignancies (solid tumors)	CFI-402257	I	Without results, Recruiting, Estimated study completion date: August 2025.	NCT05251714	
	Breast Cancer	CFI-402257 + Fulvestrant	II			
S-81694 (Completed)	Metastatic Breast Cancer	S-81694 + paclitaxel	I	The study was discontinued, and the maximum tolerated dose was not found. The use of S-81694 combined with paclitaxel does not seem to be effective in metastatic breast tumors.	NCT03411161	
	Metastatic Triple-Negative Breast Cancer	S-81694 + paclitaxel	II			
		https://www.clinicaltrials.gov/ (accessed on 2 September 2024)				

## Data Availability

Data can be shared upon request.
